# A practical study on a stepwise simulation intervention guided by video review to improve the quality of cardiopulmonary resuscitation in the pediatric emergency department

**DOI:** 10.3389/fped.2026.1873550

**Published:** 2026-07-30

**Authors:** Xiaodi Cai, Yang Chen, Qian Wang, Tingting Xue, Yanhong Zhang, Wenchao Wang, Jian Ma, Guoping Lu, Ye Cheng

**Affiliations:** Department of Critical Care Medicine, Children's Hospital of Fudan University, Shanghai, China

**Keywords:** cardiopulmonary resuscitation, *in situ* simulation, pediatric emergency, quality improvement, video review

## Abstract

**Background:**

Maintaining high-quality cardiopulmonary resuscitation (CPR) in pediatric emergency departments remains challenging. Evidence supporting integrated quality improvement strategies combining video review and *in situ* simulation is limited.

**Objective:**

To evaluate whether implementation of a video review-guided stepwise quality improvement strategy was associated with improved CPR process quality across three sequential implementation phases.

**Methods:**

This single-center before-after quality improvement study included consecutive pediatric cardiac arrest cases treated between January 2025 and February 2026. A video review-guided stepwise intervention was implemented in three phases: baseline (Group A), structural optimization (Group B), and behavioral reinforcement with monthly *in situ* simulation (Group C). The primary outcomes were chest compression fraction (CCF) and return of spontaneous circulation (ROSC).

**Results:**

Fifteen pediatric cardiac arrest cases were included (*n* = 5 per group). Median CCF increased from 80.2% (74.8–85.0) in Group A to 84.0% (83.4–88.6) in Group B and 91.3% (91.0–91.5) in Group C. An overall comparison demonstrated a significant difference among the three phases (*P* = 0.035), with *post hoc* analysis showing a significant difference between Groups A and C (*P* = 0.016). The median number of prolonged chest compression interruptions per case decreased, and role allocation improved (*P* = 0.045). No significant difference in ROSC was observed (*P* = 0.999). Airway management and patient assessment were the leading causes of prolonged interruptions.

**Conclusions:**

Implementation of a video review-guided stepwise quality improvement strategy was associated with improved CPR process quality. This approach may provide a practical framework for continuous resuscitation quality improvement and warrants further evaluation in multicenter studies.

## 1 Introduction

High-quality cardiopulmonary resuscitation (CPR) is essential for improving outcomes after pediatric cardiac arrest. Current resuscitation guidelines emphasize minimizing interruptions in chest compressions and maintaining a high chest compression fraction (CCF), which reflects the proportion of resuscitation time during which chest compressions are actively delivered (1, 2). Previous clinical studies and systematic reviews have shown that higher CCF is associated with an increased likelihood of return of spontaneous circulation (ROSC) and improved survival outcomes (3, 4). Therefore, CCF is widely used as a key process measure for evaluating CPR quality and quality improvement interventions.

Despite clear guideline recommendations, maintaining high-quality CPR in pediatric emergency departments remains challenging. Resuscitation performance may be affected by multiple interacting factors, including equipment availability, environmental constraints, workflow design, role allocation, communication efficiency, team coordination, and individual technical skills (2). These system- and team-level factors are difficult to identify through conventional debriefing alone, particularly in time-critical emergency settings.

Video review provides an objective method for evaluating real resuscitation performance. By reconstructing the resuscitation timeline, it allows detailed identification of chest compression interruptions, delays in key interventions, equipment-related problems, and communication or role-allocation deficiencies (5). In parallel, *in situ* simulation has been increasingly used to improve resuscitation performance by training multidisciplinary teams within their actual clinical environment and by exposing latent workflow and system issues (6–8).

However, previous studies have generally evaluated video review, simulation training, or CPR coaching as separate interventions (5–9). Evidence remains limited regarding how deficiencies identified through video review can be systematically translated into sequential structural optimization and sustained behavioral reinforcement in routine pediatric emergency practice. To address this gap, we developed a video review-guided stepwise quality improvement strategy consisting of baseline assessment, structural optimization, and behavioral reinforcement through monthly *in situ* simulation.

The present study aimed to evaluate whether implementation of this stepwise quality improvement strategy was associated with improvements in CPR process quality across three sequential implementation phases in a pediatric emergency department. We also sought to characterize the major contributors to prolonged chest compression interruptions identified through video review.

## 2 Methods

### 2.1 Study design and setting

This single-center quality improvement (QI) study employed a pragmatic before–after design and was conducted in the Pediatric Emergency Department of a tertiary pediatric hospital between January 2025 and February 2026. The study was reported in accordance with the Standards for Quality Improvement Reporting Excellence (SQUIRE 2.0) guidelines (10).

The study protocol was developed before implementation. Because this project evaluated routine clinical practice as a QI initiative rather than an interventional clinical trial, prospective trial registration was not performed. The studies involving human participants were reviewed and approved by the Ethics Committee of Children's Hospital of Fudan University [Approval No. Fu Er Lun Shen (2021) 431]. This study was conducted as a subproject under a larger institutional research and quality improvement program and was within the scope of the approved protocol. Written informed consent to participate in this study was provided by the participants' legal guardians.

### 2.2 Study population

Consecutive pediatric patients aged 28 days to 18 years who underwent CPR in the pediatric emergency department during the study period were screened. Eligible patients included both in-hospital cardiac arrest occurring in the emergency department and out-of-hospital cardiac arrest requiring continued resuscitation after arrival. Cases were excluded if CPR lasted less than 2 min, because reliable assessment of CPR process quality was not feasible in very short resuscitation events, or if video recordings were incomplete or technically inadequate.

No formal sample size calculation was performed because this was a pragmatic QI study including all consecutive eligible cases during the study period.

### 2.3 Video review and problem identification

The pediatric resuscitation room was equipped with a routine surveillance video system for clinical safety and departmental quality improvement. No additional cameras or recording procedures were introduced specifically for this study. All eligible resuscitations were reviewed using recordings obtained from this routine system. Videos were independently reviewed by two experienced pediatric emergency physicians who were certified American Heart Association Basic Life Support and Pediatric Advanced Life Support Faculty Instructors. Before formal review, both reviewers completed a standardized calibration session using representative resuscitation videos.

A standardized video review form developed according to pediatric resuscitation guidelines and institutional quality indicators was used to document key events, including CPR initiation, chest compression interruptions, first defibrillation, first epinephrine administration, airway management, equipment preparation, and team coordination. Chest compression depth and rate were not quantitatively analyzed because the video system did not allow reliable retrospective measurement across all phases. Therefore, CCF and interruption-related measures were selected as the main CPR process indicators.

Because the phases were implemented sequentially, reviewer blinding was not feasible. To reduce assessment bias, videos were reviewed independently, and discrepancies were resolved by consensus. Formal inter-rater reliability statistics were not prospectively calculated because consensus review was part of the predefined QI process rather than a validation study. A multidisciplinary QI core team reviewed aggregated video findings, provided feedback to frontline staff, developed structural optimization measures, and coordinated the subsequent simulation training and debriefing.

### 2.4 Stepwise intervention strategy

The study consisted of three sequential implementation phases.

#### 2.4.1 Group A: baseline phase, January-February 2025

Video review was used to identify CPR quality gaps and develop the stepwise intervention strategy.

#### 2.4.2 Group B: structural optimization phase, March-June 2025

Structural interventions included standardized resuscitation kits, CPR feedback devices, staff skills training, role assignment cards, and optimized team positioning. CPR feedback devices were introduced during this phase and were not available throughout the baseline phase. Staff skills training focused on high-quality pediatric CPR, airway management, vascular access, emergency medication preparation, feedback device use, and role-specific task execution.

#### 2.4.3 Group C: behavioral reinforcement phase, July 2025-February 2026

Monthly standardized *in situ* simulation sessions lasting approximately 30 min were conducted using predefined scripts. Each session included a pediatric cardiac arrest scenario followed by structured facilitator-led debriefing conducted by experienced institutional simulation instructors. Simulation scenarios were developed from recurrent deficiencies identified during video review and focused on airway management, equipment preparation, role allocation, closed-loop communication, and adherence to pediatric resuscitation algorithms. No additional CPR educational programs were introduced during the study period. Simulation debriefings were guided by predefined performance indicators, including prolonged chest compression interruptions, equipment preparedness, role allocation, closed-loop communication, and adherence to pediatric resuscitation algorithms.

### 2.5 Outcome measures

The primary outcomes were CCF and ROSC. CCF was defined as the proportion of total resuscitation time during which chest compressions were actively delivered:

CCF=totalchestcompressiontime/totalresuscitationtime×100%.

ROSC was defined as the restoration of spontaneous circulation with an organized cardiac rhythm accompanied by a palpable central pulse or measurable blood pressure, allowing discontinuation of chest compressions for at least 20 consecutive minutes.

Secondary outcomes included compression interruptions lasting >10 s, epinephrine administration within 5 min, time to first defibrillation if applicable, equipment preparedness, clarity of role allocation, and early establishment of an advanced airway.

### 2.6 Statistical analysis

Statistical analyses were performed using SPSS 26.0. Continuous variables were expressed as median and interquartile range, and categorical variables as frequencies and percentages. Categorical variables were compared using the chi-square test or Fisher's exact test, as appropriate, and continuous variables were compared using nonparametric tests. Outcomes were first compared across the three sequential implementation phases; when the overall comparison was statistically significant, *post hoc* pairwise comparisons were performed. A two-sided *P* value <0.05 was considered statistically significant.

No adjustment for multiple comparisons was performed because this exploratory QI study primarily aimed to evaluate process improvement rather than test confirmatory hypotheses.

### 2.7 Measures to minimize bias and confounding

Given the before–after design, several measures were used to reduce potential bias. The study was conducted in a single pediatric emergency department with a relatively stable multidisciplinary resuscitation team and a predefined QI protocol. No major changes in institutional resuscitation protocols or additional CPR educational programs occurred during the study period, as monthly *in situ* simulation replaced routine CPR training. Baseline characteristics were compared across phases. Nevertheless, residual confounding could not be completely excluded and is addressed in the Discussion.

## 3 Results

### 3.1 Baseline characteristics

During the study period, all consecutive pediatric cardiac arrest cases meeting the eligibility criteria were screened. Fifteen patients were included in the final analysis, with five cases in each implementation phase (Groups A–C). No screened cases were excluded because of CPR duration <2 min, incomplete video recordings, lack of consent for video review, or other administrative reasons.

Baseline characteristics are summarized in Table 1. No statistically significant differences were observed among the three groups in terms of age, sex, type of cardiac arrest, primary disease, prehospital CPR duration, or total CPR duration (all *p* > 0.05), indicating comparable baseline characteristics across the three sequential implementation phases.

**Table 1:** Baseline characteristics of patients.

Variable	Group A (*n* = 5)	Group B (*n* = 5)	Group C (*n* = 5)	Total (*N* = 15)	*P*
Baseline characteristics					
Age (years), median (IQR)	8.33 (0.46–10.58)	5.17 (0.17–8.67)	7.00 (5.08–13.58)	6.83 (0.67–11.25)	0.482
Sex, *n* (%)					
Male	4 (80)	4 (80)	4 (80)	12 (80)	
Female	1 (20)	1 (20)	1 (20)	3 (20)	
Type of cardiac arrest					
Asystole	5 (100)	5 (100)	5 (100)	15 (100)	
Primary disease, *n* (%)					
Neurological (brain tumor, cerebral palsy)	2 (40)	2 (40)	2 (40)	6 (40)	
Accidental injury	3 (60)	2 (40)	3 (60)	8 (53.3)	
Others	0	1 (20)	0	1 (6.7)	
Prehospital CPR >15 min, *n* (%)	2 (40)	2 (40)	4 (80)	8 (53.3)	0.317
CPR duration (min), median (IQR)	26 (16–32)	38 (35–46)	36 (33–50)	33 (26–46)	0.056

All included patients presented with asystole as the initial cardiac rhythm, and therefore no patient required defibrillation during resuscitation.

### 3.2 Primary outcomes

Primary outcomes are presented in Table 2.

**Table 2:** Primary outcomes and process quality indicators.

Variable	Group A (*n* = 5)	Group B (*n* = 5)	Group C (*n* = 5)	Total (*N* = 15)	*P*
Primary outcomes					
Chest compression fraction (CCF, %), median (IQR)	80.2 (74.8–85.0)	84.0 (83.4–88.6)	91.3 (91.0–91.5)	86.6 (80.2–91.3)	0.035*
Return of spontaneous circulation (ROSC), *n* (%)	3 (60)	2 (40)	2 (40)	7 (46.7)	0.999
Process quality indicators, *n* (%)					
Complete preparation of resuscitation equipment	0 (0)	3 (60)	3 (60)	6 (40)	
Timely arrival of personnel after activation	3 (60)	3 (60)	5 (100)	11 (73.3)	
ECG monitoring (within 2 min)	4 (80)	2 (40)	5 (100)	11 (73.3)	
Early initiation of CPR	3 (60)	4 (80)	5 (100)	12 (80)	
Defibrillation**	–	–	–	–	
Establishment of IV/IO access within 5 min	5 (100)	3 (60)	5 (100)	13 (86.7)	
Compression interruption >10 s (occurrence)	5 (100)	3 (60)	4 (80)	12 (80)	
Use of feedback devices	0 (0)	3 (60)	3 (60)	6 (40)	
Appropriate bag-valve-mask ventilation	4 (80)	4 (80)	5 (100)	13 (86.7)	
Early advanced airway (within 5 min)	4 (80)	5 (100)	5 (100)	14 (93.3)	
Appropriate compression–ventilation ratio during CPR	2 (40)	1 (20)	3 (60)	6 (40)	
Timely epinephrine administration (within 5 min)	5 (100)	3 (60)	5 (100)	13 (86.7)	
Clear instructions and closed-loop communication	4 (80)	3 (60)	5 (100)	12 (80)	
Clear and appropriate role allocation	0 (0)	2 (40)	3 (60)	5 (33.3)	0.045

* Comparison of CCF among groups: Kruskal–Wallis test showed a statistically significant difference (H = 6.72, *P* = 0.035). Pairwise comparisons (Mann–Whitney U test): A vs. B, *P* = 0.31; A vs. C, *P* = 0.016; B vs. C, *P* = 0.056.

** Defibrillation was not applicable because all included patients presented with asystole.

Median CCF increased across the three implementation phases, from 80.2% (74.8–85.0) in Group A to 84.0% (83.4–88.6) in Group B and 91.3% (91.0–91.5) in Group C. A statistically significant overall difference was observed among the three implementation phases (Kruskal–Wallis test, H = 6.72, *P* = 0.035). Pairwise comparisons demonstrated a statistically significant difference between Groups A and C (*P* = 0.016), whereas comparisons between Groups A and B (*P* = 0.31) and between Groups B and C (*P* = 0.056) did not reach statistical significance.

The proportion of cases achieving CCF ≥90% appeared higher in the later implementation phases than in the baseline phase, although this indicator was interpreted descriptively because of the small sample size.

ROSC occurred in 3 of 5 patients (60%) in Group A, 2 of 5 patients (40%) in Group B, and 2 of 5 patients (40%) in Group C, with no statistically significant difference among the three groups (*P* = 0.999).

### 3.3 CPR process quality indicators

Complete preparation of resuscitation equipment was documented in 0%, 60%, and 60% of cases in Groups A, B, and C, respectively. Timely arrival of personnel after emergency activation occurred in 60%, 60%, and 100% of cases across the three phases. Early initiation of CPR was observed in 60%, 80%, and 100% of cases, respectively.

Early establishment of an advanced airway within 5 min was achieved in 80% of patients in Group A and in all patients in Groups B and C. Timely epinephrine administration within 5 min occurred in 100%, 60%, and 100% of cases, respectively.

Clear role allocation increased from 0% in Group A to 40% in Group B and 60% in Group C, representing the only process indicator that reached statistical significance (*P* = 0.045).

The occurrence of chest compression interruptions lasting longer than 10 s was recorded in all cases in Group A, three cases in Group B, and four cases in Group C.

### 3.4 Causes of chest compression interruptions

The causes of chest compression interruptions are summarized in Table 3.

**Table 3:** Analysis of causes of chest compression interruptions.

Variable	Group A (*n* = 5)	Group B (*n* = 5)	Group C (*n* = 5)	Total
Number of compression interruptions >10 s per case	4 (3–5)	2 (1–3)	0–1	
Causes of interruptions (*n*)				
Airway management (intubation/adjustment)	6	4	2	12
Patient assessment (pulse/rhythm check)	4	2	1	7
Equipment-related procedures (e.g., chest x-ray)	3	2	1	6
Rescuer switching	2	1	0	3
Team communication delay or unclear commands	2	1	0	3

Airway management, including tracheal intubation and adjustment of airway devices, accounted for the largest number of prolonged interruptions (12 events). Patient assessment, including pulse and rhythm evaluation, accounted for seven interruptions, followed by equipment-related procedures (6 events).

The number of chest compression interruptions lasting longer than 10 s decreased from 4 (3–5) per case in Group A to 2 (1–3) in Group B and 0–1 per case in Group C.

Fewer interruption events were attributed to rescuer switching and communication-related delays across all study phases.

## 4 Discussion

This study developed and evaluated a stepwise quality improvement model integrating video review, structural optimization, and behavioral reinforcement through *in situ* simulation. The main finding was that implementation of this integrated strategy was associated with improvements in several CPR process quality indicators, particularly CCF, role allocation, equipment preparedness, and prolonged chest compression interruptions. However, no statistically significant difference in ROSC was observed among the three groups. These findings suggest that this stepwise approach may improve CPR process quality in the pediatric emergency setting, although its impact on patient-centered outcomes remains uncertain.

One of the major strengths of the present study is the integration of video review into a continuous quality improvement framework. Unlike conventional debriefing, which relies largely on participant recall, video review provides an objective and reproducible assessment of resuscitation performance. In this study, detailed timeline analysis enabled the identification of recurrent factors affecting CPR quality, particularly airway management, patient assessment, team coordination, equipment preparedness, and communication failures. These findings are consistent with previous studies (5, 7, 11) showing that video-assisted review can identify latent safety threats and that interruptions during airway procedures and deficiencies in team dynamics are major contributors to reduced CPR quality. Importantly, the video review findings were subsequently translated into targeted organizational interventions and simulation scenarios, thereby establishing a direct link between performance evaluation and quality improvement.

Importantly, this study moves beyond single-component interventions by implementing a stepwise strategy that links problem identification with targeted system-level and behavioral interventions. Structural optimization addressed system-related barriers, such as equipment availability, team layout, role assignment, and technical preparation, which are often overlooked but important for high-quality resuscitation. Behavioral reinforcement through regular *in situ* simulation was then used to reinforce standardized workflows, team coordination, communication, and adherence to resuscitation protocols. Simulation scenarios were designed based on high-frequency issues identified through video review, enabling teams to repeatedly practice in the real clinical environment. Although CCF increased further from Group B to Group C and the median number of prolonged interruptions continued to decrease, the difference between Groups B and C did not reach statistical significance. Therefore, the present study cannot conclude that simulation training independently improved CPR quality. Because structural optimization and simulation were implemented sequentially as components of the same quality improvement strategy rather than as independent interventions, the specific contribution of simulation could not be isolated. Rather, simulation may have contributed to consolidating and maintaining the improvements achieved through the broader stepwise quality improvement strategy, which is consistent with findings from simulation studies (7, 8, 12, 13).

This integrated approach represents a closed-loop quality improvement process, in which performance gaps identified through video review are systematically addressed and reinforced through targeted interventions. Compared with previous studies (5, 6) that evaluated video review or simulation training in isolation, the present model provides a practical framework for linking objective performance assessment with system-level optimization and team-based behavioral reinforcement. In addition, video review identified airway management as the leading cause of prolonged chest compression interruptions, followed by patient assessment and equipment-related procedures. These findings provided practical targets for structural changes and simulation training, including equipment organization, task allocation, airway-related workflow, and closed-loop communication.

Despite improvements in CPR process quality, no statistically significant difference in ROSC was observed among the groups. This finding may be attributed to several factors. First, the study included only fifteen patients and was not statistically powered to detect differences in clinical outcomes. Second, more than half of the patients received prolonged prehospital CPR before arrival, which may have limited the effect of subsequent in-hospital CPR quality improvement on immediate outcomes. Third, all included patients presented with asystole, a non-shockable rhythm associated with poor prognosis, and no defibrillation events occurred. Therefore, improvements in CPR process measures may not necessarily translate into measurable improvements in ROSC in such a small and clinically severe cohort. Nevertheless, improvements in CPR quality remain clinically important, as high-quality CPR has been associated with improved survival and neurological outcomes in previous studies (9, 13).

From a clinical perspective, this study provides a practical model for continuous CPR quality improvement in a real-world pediatric emergency setting. The combination of video review and *in situ* simulation allows for ongoing performance monitoring, feedback, and reinforcement using existing institutional resources. This approach may be particularly useful for emergency departments seeking to improve resuscitation workflow, team coordination, and adherence to standardized processes. However, given the single-center design and small sample size, the applicability of this model to other institutions should be further evaluated.

This study has several limitations. First, it was conducted at a single center with a small sample size, which limited statistical power and generalizability, especially for patient-centered outcomes such as ROSC. Second, the before–after design may be affected by temporal confounding and the Hawthorne effect, although staffing, institutional protocols, and concurrent CPR education remained stable during the study period. Third, formal inter-rater reliability statistics were not prospectively calculated, although reviewer calibration and consensus procedures were used. Finally, long-term outcomes, including neurological outcomes, were not assessed. Future multicenter studies with larger sample sizes and more robust designs are warranted to validate these findings.

## 5 Conclusion

This study supports the feasibility of implementing a video review-guided stepwise quality improvement strategy in a pediatric emergency department. The intervention was associated with improvements in CPR process quality, particularly chest compression fraction, equipment preparedness, role allocation, and workflow performance. Although no significant improvement in ROSC was observed, this model may provide a practical framework for continuous resuscitation quality improvement. Larger multicenter studies are required to validate these findings and evaluate their impact on patient-centered outcomes.

## Data Availability

The original contributions presented in the study are included in the article/Supplementary Material, further inquiries can be directed to the corresponding author.
