# Supplementary material for: A practical study on a stepwise simulation intervention guided by video review to improve the quality of cardiopulmonary resuscitation in the pediatric emergency department
Source: Front Pediatr. 2026 Jul 30;14:1873550. doi: 10.3389/fped.2026.1873550 (PMC13468511; doi:10.3389/fped.2026.1873550)
Supplement: Supplementary file 1 [file Supplementaryfile1.docx]

Appendix 1 Standardized Scoring Form

| CPR date:  Leader:  Nursing Leader:  nurse:  docter:  Number of doctors involved in resuscitation; Number of nurses involved in resuscitation | Video review date:  Team members check-in:  BLS Instructor ：  PALS Instructor  BLS TCF  PALS TCF: | | |
| --- | --- | --- | --- |
| Quality indicators of cardiopulmonary resuscitation | Is there a problem? | | |
|  | **Yes** | **No** | concrete issue |
| Rescue equipment/items are fully prepared |  |  |  |
| □ Complete medication |  |  |  |
| □ Team call in time / Arrive on time |  |  |  |
| □ Reasonable role allocation |  |  |  |
| □ Clear role responsibilities |  |  |  |
| **recurrence** | **Yes** | **No** | Specific issues that could not be addressed |
| □Complete monitoring within 2 minutes and prepare for defibrillation as needed. |  |  |  |
| □Start chest compressions as early as possible |  |  |  |
| □ IV/IO activation time |  |  |  |
| □ Chest compressions interrupted for <10 seconds (see below for reasons of failure) |  |  |  |
| □ A Pressing the personnel queue  □ B Personnel rotation for pressing  □ C defibrillation  □ D Airway Management  □ E Patient Condition Assessment  □ F other |  |  |  |
| □ Use feedback device () |  |  |  |
| High-Quality Cardiopulmonary Resuscitation  CCF |  |  |  |
| air flue | **Yes** | **No** | Specific issues that could not be addressed |
| □ Appropriate breathing ballon ventilation device (BVM) |  |  |  |
| □ Early preparation for endotracheal intubation; |  |  |  |
| □ Is endotracheal intubation performed? |  |  |  |
| □ During chest compressions or ventilation, minimize interruptions to facilitate intubation. |  |  |  |
| □ Appropriate ventilation compression ratio |  |  |  |
| **Advanced Support** | **Yes** | **No** | Specific issues that could not be addressed |
| □Administer epinephrine as early as possible (within 5 minutes) |  |  |  |
| □ Perform the assessment (pulse /rhythm every 2 minutes) |  |  |  |
| □Is bedside ultrasound |  |  |  |
| communicate | **Yes** | **No** | Specific issues that could not be addressed |
| Clear instructions and closed-loop communication |  |  |  |

Summary: Focus on CCF and key aspects (venue, equipment, teamwork, individual skills, and workflow), and provide at least three points.
