# Supplementary material for: A practical study on a stepwise simulation intervention guided by video review to improve the quality of cardiopulmonary resuscitation in the pediatric emergency department
Source: Front Pediatr. 2026 Jul 30;14:1873550. doi: 10.3389/fped.2026.1873550 (PMC13468511; doi:10.3389/fped.2026.1873550)
Supplement: Supplementary file 3 [file Supplementaryfile3.docx]

Appendix 3 In-Situ Simulation Scenario for Cardiopulmonary Resuscitation in the Emergency Resuscitation Room

In-situ CPR Simulation Script in the Emergency Resuscitation Room

Objective:

TeamSTEPPS elements

Assessment of quality improvement points

1. Location: Resuscitation bay preparation
2. Equipment and personnel preparation: Crash cart, metronome, call-out, etc.
3. Personnel skills: Elements of high-quality CPR (CPR feedback device)
4. Team collaboration: Role distribution - flow chart, whether CPR is paused during intubation coordination
5. Evaluation of the Nursing Leader role and the Medical Leader role

Duration: 10-12 minutes simulation + 10 minutes debriefing

Location: Emergency Resuscitation Room

## Roles:

1. Medical Leader: Responsible for directing the resuscitation efforts, performing key procedures and making decisions. (Position 7)
2. Nursing Leader: Prepares emergency equipment and medications, assists the leader, guides and coordinates the CPR team and quality. (Nursing Team Lead)
3. 2-3 Nurses
4. 2-3 Doctors: Emergency Medicine Chief Resident, Resident from the Observation Unit or Emergency Ward, one Emergency Medicine Doctor B
5. Patient: A child reported to be unresponsive, CPR in progress en route.

## Scenario

1. Phone pre-notification (15:20): An unresponsive infant in cardiac arrest, CPR in progress, not intubated. EMS estimates arrival in 10 minutes.
2. 10 minutes later: The patient (simulated infant) is brought into the resuscitation room by a simulated patient (SP) performing incorrect CPR.

## Script Flow

## Debriefing

Tool: Debriefing Form
