# Supplementary material for: A practical study on a stepwise simulation intervention guided by video review to improve the quality of cardiopulmonary resuscitation in the pediatric emergency department
Source: Front Pediatr. 2026 Jul 30;14:1873550. doi: 10.3389/fped.2026.1873550 (PMC13468511; doi:10.3389/fped.2026.1873550)
Supplement: Supplementary file 4 [file Supplementaryfile2.docx]

Appendix 2 Resuscitation Station Layout
